# Prevalence, Identification and Mycotoxigenic Potential of Fungi in Common Spices Used in Local Malaysian Cuisines

**DOI:** 10.3390/foods11172548

**Published:** 2022-08-23

**Authors:** Syamilah Nordin, Nurul Afifah Samsudin, Effarizah Mohd Esah, Latiffah Zakaria, Jinap Selamat, Mohd Azuar Hamizan Rahman, Norlia Mahror

**Affiliations:** 1 Food Technology Division, School of Industrial Technology, Universiti Sains Malaysia (USM), Gelugor 11800, Pulau Pinang, Malaysia; 2 School of Biological Sciences, Universiti Sains Malaysia (USM), Gelugor 11800, Pulau Pinang, Malaysia; 3 Laboratory of Food Safety and Food Integrity, Institute of Tropical Agriculture and Food Security, Universiti Putra Malaysia (UPM), Serdang 43400, Selangor, Malaysia; 4 Department of Food Science, Faculty of Food Science and Technology, Universiti Putra Malaysia (UPM), Serdang 43400, Selangor, Malaysia

**Keywords:** food safety and security, spices, fungi, *Aspergillus* spp., mycotoxins

## Abstract

Spices are widely used in various cuisines in Malaysia to enhance the flavour and aroma. However, spices are susceptible to fungal infection, leading to mycotoxin contamination if the storage conditions are favourable for fungal growth. Thus, this study aimed to identify fungal species in spices commonly used in local Malaysian cuisines and determine their prevalence and mycotoxigenic potential. A total of 110 spice samples consisting of cumin, fennel, coriander, peppers (black pepper and white pepper), chillies (dried chilli, chilli paste and chilli powder), cinnamon, star anise, cloves, curry powder and korma powder were randomly purchased from retail markets in Penang. The samples were analysed for the total fungal count (ground spices) and the incidence of fungal infection (whole spices). The fungal species isolated from spices were identified based on morphological and molecular approaches, and the mycotoxigenic potential was determined using the Coconut Cream Agar method. The results showed that coriander seeds (ground) recorded the highest total fungal count (ADM 3.08 log CFU/g; DG18 3.14 log CFU/g), while black pepper (whole) recorded the highest incidence of fungal infection (94%). Interestingly, star anise and cloves were free from fungal contamination. The mycotoxigenic fungi of *A. flavus* and *A. niger* recorded the highest isolation frequency in ground and whole spices. These findings indicate the risk of mycotoxin exposure to consumers due to the high consumption of spices in local Malaysian cuisine.

## 1. Introduction

Spices are derived from different parts of plants such as seeds, barks, flowers, roots, leaves and stigmas [1]. Globally, spices have been widely used in various cuisines since ancient times to enhance flavour, aroma and colouring and also for use as preservatives [2]. Spices are sold in the market as a whole, grounded or mixed spice. In Malaysia, a variety of popular cuisines use spices as the main ingredients or condiments. Some popular cuisines in Malaysia include *nasi kandar*, *rendang*, *nasi lemak*, *sambal tumis*, curry dishes and diverse types of soup. Spices such as chilli, cinnamon, cloves, coriander seeds, peppers and star anise are popular spices used in preparing those cuisines.

Nevertheless, spices are prone to be contaminated with various contaminants, including mycotoxigenic fungi and mycotoxins [3]. The susceptibility of other foodstuffs such as nuts, cereals, peanuts, maize and rice was also reported worldwide [4,5,6,7]. Mycotoxigenic fungi are able to produce naturally occurring toxic metabolites in these commodities known as mycotoxins if the storage conditions are favourable to the fungal growth [8]. Improper handling and practices during post-harvest and the transportation from the field to the market will also cause fungal growth and mycotoxin production. According to Afsah-Hejri et al. [9], the tropical climates in Malaysia throughout the year, with high temperatures (28–31 °C) and a high relative humidity (70–90%), are conducive to fungal growth and mycotoxin production.

Some fungal genera that can contaminate dried commodities and are capable of producing mycotoxins are *Aspergillus*, *Penicillium* and *Fusarium* [9]. *Fusarium* is known as field fungi since it is commonly found in the crops during pre-harvest. In contrast, the *Aspergillus* and *Penicillium* are considered as storage fungi due to their ability to grow in stored crops and invade the grains or seed during storage [10,11,12]. *Aspergillus* and *Penicillium* are known as xerophilic fungi that generally occur in low-moisture conditions [13]. Mycotoxins can cause illness and are detrimental to human health due to chronic exposure [14]. Among all the identified mycotoxins, the International Agency for Research on Cancer [15] has reported that aflatoxin (AF) and ochratoxin A (OTA) are the most harmful mycotoxins to humans. *A*. *flavus* is the main producer of AFB_1_ and AFB_2_, while other species such as *A*. *niger*, *A*. *carbonarius* and *Penicillium verrucosum* are able to produce OTA [16,17,18].

The occurrence of mycotoxins and fungi in dried commodities, including spices, has been reported in various countries, including Malaysia [1,9,13,19,20,21]. However, the mycological studies of spices from the retail market, especially in Malaysia, are very limited. Several researchers have reported on mycotoxins in spices from Malaysia, but only a few of them studied the microbiological aspect [9,22,23]. For example, Ali et al. [24] reported the occurrence of AF and OTA from 34 spices in Malaysia, ranging from 0.01–9.34 µg/kg and 0.14–20.40 µg/kg, respectively. Another study by Jalili et al. [22] pointed out that 65% and 81% of commercial dried chilies were contaminated with AF and OTA, respectively. A recent study by Ahmad Zaidi et al. [19] reported that AF and OTA were detected in chilli products such as chilli paste and chilli sauce from different manufacturers. The authors revealed that samples from the small-scale manufacturers contain high levels of total fungal load compared to those from the large-scale manufacturers. Hence, it is essential to determine the presence of fungi in spices, as the high consumption of food containing spices may lead to high mycotoxin exposure to the consumers and thereby affect the consumers’ health. Therefore, this study aims to determine the prevalence of mycotoxigenic fungi in spices used in local Malaysian cuisines, to identify the species based on the morphological, molecular and biochemical characteristics and to determine the mycotoxigenic potential of the fungal isolates.

## 2. Materials and Methods

### 2.1. Samples

A total of 110 packed and unpacked spice samples corresponding to 13 types of spice samples were purchased randomly from retail markets in Penang. Packed samples were packed in polyethylene bags, whereas unpacked samples were taken from the market in plastic containers or gunny bags. The collected spices were cumin, fennel, coriander, peppers (black pepper, white pepper, dried chilli, chilli paste and chilli powder), cinnamon, star anise, cloves, curry powder and korma powder. Each spice consists of five whole spice and ground spice samples. All samples were kept in a storage box at room temperature for a maximum of one week until analysis.

### 2.2. Chemicals and Reagents

The PCR Master Mix and the ITS 1 and ITS 4 primers for PCR reaction were purchased from a local supplier (Apical Scientific, Seri Kembangan, Malaysia).

### 2.3. Water Activity Analysis

The water activity of each spice was determined in duplicate using a water activity meter (Aqualab PRE, Decagon Devices Inc., Pullman, WA, USA).

### 2.4. Prevalence of Fungi in Spices

*Aspergillus* Differentiation Medium (ADM) (HiMedia, Mumbai, India) and Dichloran-glycerol agar (DG18) (HiMedia, Mumbai, India) were used to determine the prevalence of fungi in the spices. ADM was supplemented with one vial of chloramphenicol selective supplement (HiMedia, Mumbai, India) to suppress the spreading fungi from overgrowth on the plates.

The total fungal counts in ground spices were analysed using the dilution plating method according to Pitt & Hocking [25], with a slight modification. The first dilution was obtained by homogenising 25 g of ground samples in 225 mL of 0.1% Buffered Peptone Water (BPW) (HiMedia, Mumbai, India) using a stomacher (Seward, Worthing, UK). Then, 1 mL aliquot was transferred into a universal bottle containing 9 mL of 0.1% BPW, yielding a 10^−2^ dilution. Subsequently, 0.1 mL aliquot of the 10^−1^ and 10^−2^ dilution were inoculated and evenly spread onto the DG18 and ADM plates. The inoculation was carried out in duplicate agar plates for each spice samples. The inoculated agar plates were incubated at 30 °C for 5 days. The total colonies were counted, and the results were expressed in colony-forming units (CFU/g).

Total fungal counts for whole spices were determined using the direct plating method according to Pitt & Hocking [25]. By using a sterile forceps, 6 to 12 whole spice samples were plated directly onto the DG18 agar, depending on its size. The particulates were plated at a 2–3 cm distance from one another. Subsequently, the plates were incubated in an incubator at 30 °C for 5 days. The number of infected particles was counted and expressed as a percentage.

### 2.5. Morphological and Molecular Identification

The mixed fungal culture on the DG18 agar was isolated using a hook and transferred aseptically onto Potato Dextrose Agar (PDA) (HiMedia, Mumbai, India) plates to obtain the pure culture of fungi. The PDA plates were incubated for 2–5 days at 30 °C. After a pure culture was obtained, it was transferred to the Malt Extract Agar (MEA) (HiMedia, Mumbai, India) and Czapek Yeast Extract (CYA) (HiMedia, Mumbai, India) agar for identification, as described by Klich [26]. The plates were incubated for 5–7 days at 30 °C [25]. The morphological characteristics of the isolates were observed and recorded.

For molecular identification, 110 isolated strains were grouped according to their morphological characteristics, and 24 representative isolates were chosen for molecular identification. The isolates were grown in potato dextrose broth (PDB) for 3–7 days. The mycelia were harvested on Whatman 2.0 filter paper, dried and ground into fine powder using liquid nitrogen. The deoxyribonucleic acid (DNA) of the isolates was extracted using the Dneasy Plant Mini Kit (QIAGEN, Hilden, Germany) according to the manufacturer’s instructions.

The ITS region of the ribosomal DNA was amplified using a set of universal primers, ITS1 and ITS4 [27]. Each PCR reaction was performed using 25 µL of PCR Master Mix 2X, which consists of 5 µL of both forward (ITS1) and reverse (ITS4) primers, 2 µL DNA template and 13 µL H_2_O. The thermocycler was set for 30 cycles: initial denaturation and denaturation at 95 °C for 1 min, annealing at 61 °C for 1 min, elongation at 72 °C for 1 min and final elongation at 72 °C for 2 min. The PCR products were sent to a local service provider (1st Base, Seri Kembangan, Malaysia) for sequencing.

The ClustalW in Mo thelecular Evolution and Genetic Analysis (MEGA 7) software [28] was used to obtain the consensus sequences by aligning and editing the forward and reverse sequences. Then, the consensus sequences were compared with the existing sequences in the GenBank database (http://www.ncbi.nlm.nih.gov, accessed on 1 July 2022) using the Basic Local Alignment Search Tool (BLAST). The similarity (99–100%) between the query and the existing sequence from the BLAST search was used to identify the isolates.

### 2.6. Mycotoxigenic Potential of Fungal Isolates

The mycotoxigenic potential of the fungal isolates was determined using coconut cream agar (CCA) following a method described by Norlia et al. [29] for AF and by Zhang et al. [30] for OTA, with a slight modification. A total of 200 mL of commercial coconut milk (M&S brand, Kuala Lumpur, Malaysia) was diluted in 1000 mL distilled water. Subsequently, 24 g of bacteriological agar was added and autoclaved for 15 min at 121 °C. The fungal isolates were inoculated at the center of CCA and incubated for 10 days at 30 °C. The isolates were observed under UV light (365 nm). AF- and OTA-producing isolates showed green fluorescence on the reverse sides of the plates.

## 3. Results

### 3.1. Water Activity

The average value of water activity (a_w_) was 0.607 and 0.642 for the ground and whole spice samples, respectively. The highest a_w_ for ground spices was korma powder (0.643), while the lowest was curry powder (0.500). For whole spices, the highest a_w_ was coriander seeds (0.711), and the lowest was cloves (0.601).

### 3.2. Prevalence of Fungi in Spices

Table 1 presents the total fungal count for different types of spices cultured on the ADM and DG18 agar. The total fungal count on the ADM and DG18 agar varied from 0.71 to 3.51 log CFU/g and from 0.45 to 3.14 log CFU/g, respectively. ADM agar is used to distinguish the aflatoxin-producing fungi from other *Aspergillus* spp. The aflatoxin-producing fungi will produce a bright yellow-orange colour at the reverse side of the colonies, differentiating the fungi from other species. The development of colour is due to the reaction of the aspergillic acid from the fungi with the ferric ammonium citrate contained in the ADM [31]. DG18 is mainly used to enumerate the total fungal count, including *Penicillium* and *Fusarium*, which are commonly present in dried commodities [19,32].

In general, coriander seeds, curry powder, korma powder and dried chilli were heavily contaminated by fungi, ranging from 1.67 to 3.51 log CFU/g. Considering all the samples, coriander seeds were the most contaminated spices (ADM 3.08 log CFU/g; DG18 3.14 log CFU/g), followed by curry powder (ADM 3.17 log CFU/g; DG18 2.80 log CFU/g), korma powder (ADM 2.59 log CFU/g; DG18 2.65 log CFU/g) and dried chilli (ADM 3.51 log CFU/g; DG18 1.67 log CFU/g). Fennel, cumin, black pepper and chilli powder were considerably high in fungal counts, ranging from 1.95 to 2.59 log CFU/g. However, low fungal counts were observed in white pepper, cinnamon and chilli paste samples, ranging from 0.45 to 2.30 log CFU/g. *A. flavus* and *A. niger* were found to be the most isolated species from these spices. *A. flavus* was abundant in chilli powder (76.3%), followed by dried chilli (45.5%), white pepper (40%) and coriander (31.6%). *A. niger* was mostly isolated from korma powder (38%), dried chilli (25.8%) and curry powder (24.2%). A variety of fungal species were present in coriander samples, namely, *A. amstelodami* (4.21%), *A. chevalieri* (1.1%), *A. flavus* (31.6%), *A niger* (10.5%), *A. sydowii* (1.1%), *A. tamarii* (1.1%), *A. terreus* (2.1%), *P. chrysogenum* (10.5%) and *P. citrinum* (1.1%). The chilli paste samples showed the lowest counts, with 0.71 and 0.45 log CFU/g in ADM and DG18, respectively. The chilli paste was the least contaminated, with only two species, which were *A. niger* (3.3%) and *A. terreus* (1.2%).

The fungal infection from the whole spice samples was recorded in terms of percentages, as shown in Table 2. This study found that black pepper was the most contaminated sample, ranging from 70% to 100%, followed by coriander seeds (92.4%), white pepper (83.2%) and cumin (63.8%). The least contaminated spices were observed in fennel (52.0%) and cinnamon (30.6%). *A. flavus* was predominant in white pepper (70.0%) and black pepper (34.0%). Meanwhile, *A. niger* was abundant in fennel (33.3%), cinnamon (33.3%) and black pepper (27.7%). Among other spices, cumin was contaminated with a variety of fungal species such as *A. amstelodami* (8.6%), *A. chevalieri* (2.9%), *A. flavus* (2.9%), *A. niger* (5.7%), *A. sydowii* (20%), *P. chrysogenum* (5.7%), *Chaetomium jabalpurense* (5.7%) and *Cladosporium tenuissimum* (2.9%). Interestingly, cloves and star anise were free from fungal infection. After several attempts to plate different cloves and star anise samples, no visible fungi from all these samples were observed.

### 3.3. Morphological and Molecular Identification

**Figure 1:**
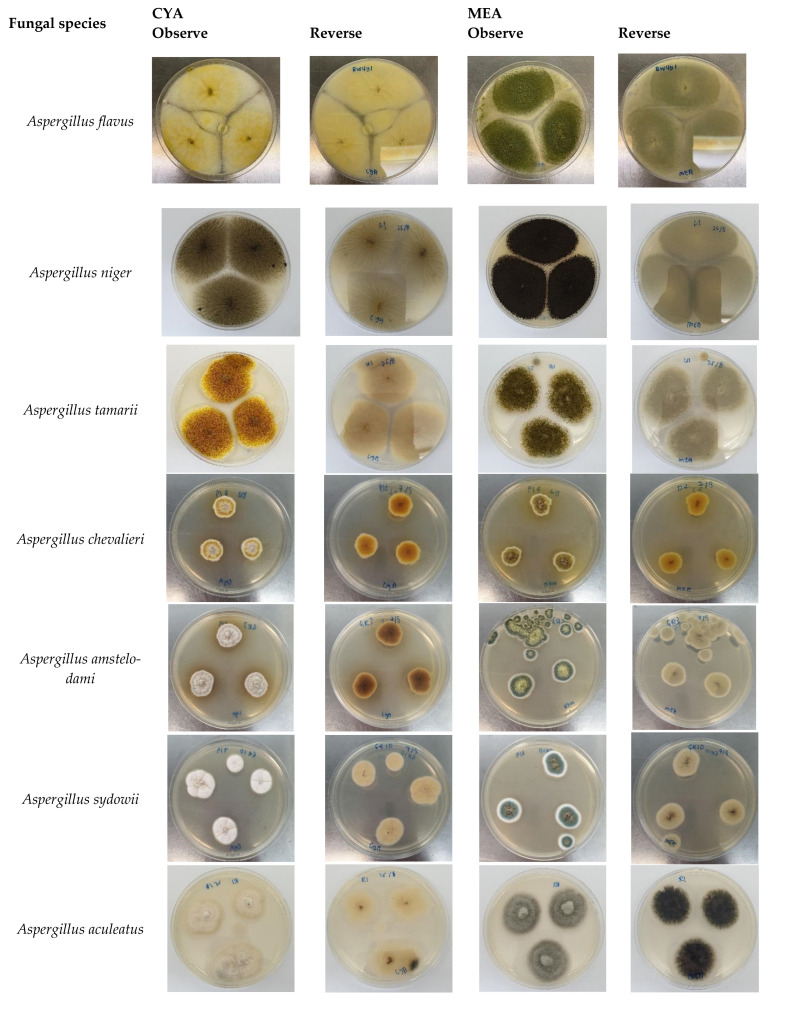

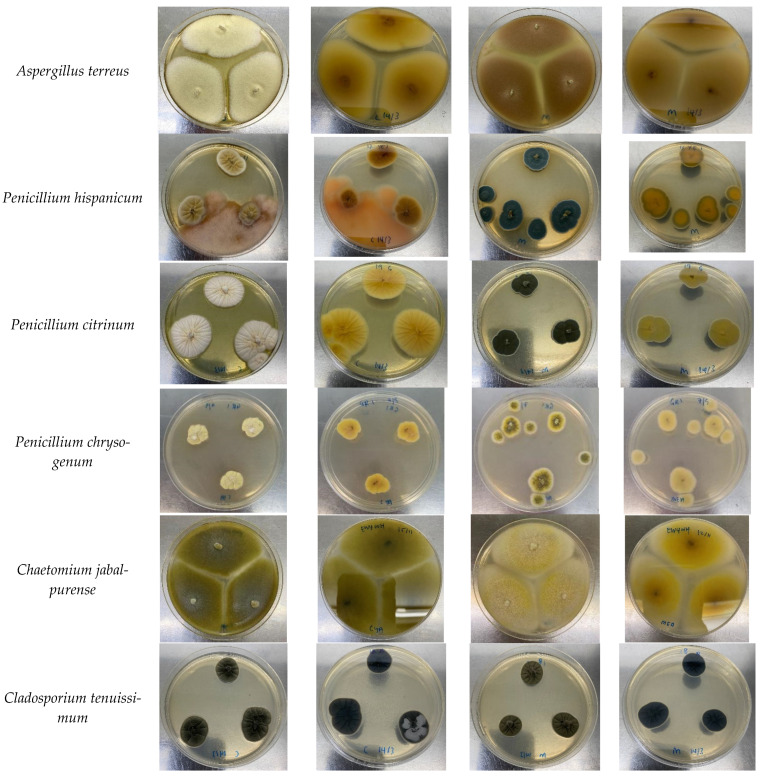
Morphological characteristics of fungal isolates on CYA and MEA after 5–7 days incubation.

The identification of fungal species was determined morphologically and molecularly. Figure 1 shows the colony morphology of the fungal species identified in this study. The molecular identification based on the rDNA ITS region showed 99% to 100% similarity with the existing sequences in the GenBank, as shown in Table 3. Thirteen fungal species were identified, which belong to the genera *Aspergillus*, *Penicillium*, *Chaetomium* and *Cladosporium*. The fungal species include *A. aculeatus*, *A. amstelodami*, *A. chevalieri*, *A. flavus*, *A. niger*, *A. sydowii*, *A. tamarii*, *A. terreus*, *P. chrysogenum*, *P. citrinum*, *P. hispanicum*, *Chaetomium jabalpurense* and *Cladosporium tenuissimum*.

The morphology of the fungi was in agreement with Klich [26]. *A. flavus* was predetermined on ADM plates, as it produced a bright yellow-orange reverse, indicating the presence of *A. flavus*. *A. flavus* on CYA and MEA was 50–60 mm in diameter after 5 days of incubation at 30 °C. On CYA, the conidia were yellow with white mycelium. The colony texture was floccose, and it had a pale yellow reverse. *A. flavus* on MEA had olive conidia with inconspicuous white mycelium. *A. niger* was 55–70 mm in diameter after being incubated for 5 days at 30 °C and grown densely on CYA plates. The conidia were black to very dark brown and had an uncoloured reverse. On MEA, the colony was black, had a thick floccose on the colonies and was thin at the edges.

*A. chevalieri* (formerly known as *Eurotium chevalieri*) was identified based on the yellow colonies on CYA and MEA. Both plates had pale yellow mycelium and a yellow reverse. *A. chevalieri* was 15–20 mm in diameter after incubation for 5 days. *A. tamarii* was 40–45 mm in diameter after 5 days of incubation at 30 °C on CYA. The colony was deep yellowish-brown with white mycelium. On MEA, the colonies’ diameter was 35–45 mm, and they were olive-brown with inconspicuous white mycelium. The vesical shape was globose. On CYA, the diameter of *A. sydowii* after 5 days of incubation was around 10–18 mm. *A. amstelodami* (formely known as *Eurotium amstelodami*) has green-yellow conidia on MEA and an uncoloured reverse. On CYA, *A*. *amstelodami* was in white dense mounds with a dark brown reverse. *A. amstelodami* grew to 17–24 mm in diameter on CYA and MEA.

*A. terreus* on CYA was in pale yellow conidia with white mycelium. The reverse colour was yellow. *A. terreus* on CYA and MEA had the same growth rate—50–65 mm after 5 days of incubation. On MEA, *A. terreus* was pale orange with inconspicuous white mycelium. The reverse colour was almost similar to that on CYA but in a darker colour.

### 3.4. Mycotoxigenic Potential of Fungal Isolates

**Figure 2:**
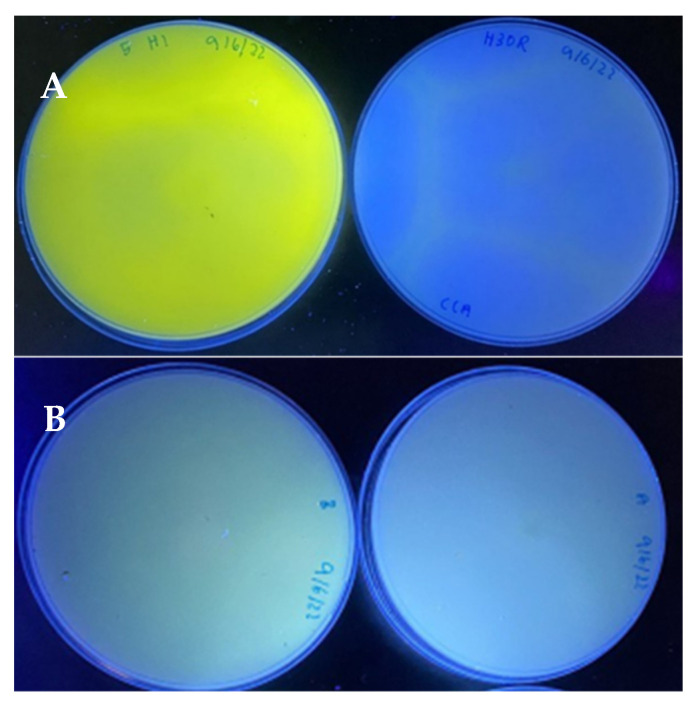
Green fluorescence (left side) was detected on the reverse colony of (**A**) *A. flavus* and (**B**) *A. niger*, which were able to produce AF and OTA on CCA, respectively, while no fluorescence was detected on the non-producer isolates (right side). The fluorescence was observed under UV light (365 nm) after 10 days of incubation.

The representative fungal isolates were tested for their ability to produce mycotoxins (AF and OTA), as shown in Table 3. The isolates of *A. flavus* and *A. niger* showed varying intensities of green fluorescence under UV light (365 nm), while the other species did not fluoresce. Figure 2 shows the intensity of green fluorescence on *A. flavus* and *A. niger* on the CCA agar, indicating the ability of these species to produce AF and OTA, respectively. The presence of these mycotoxigenic fungi has confirmed the potential risk of AF and OTA contamination in spices.

## 4. Discussion

Spices are susceptible to fungal growth in their favourable conditions. This study provided important knowledge regarding the fungal contamination in various spices used in Malaysia. From this study, the a_w_ levels were low (0.500–0.711), indicating that the spices have sufficiently dried prior to storage. Due to the low a_w_ levels, the dominant group of fungi in the samples was xerophilic fungi, which favour low-moisture conditions. The results showed that fennel, coriander seeds, black pepper, curry powder, chilli powder, dried chilli, cumin and korma powder were highly contaminated with fungi. Our finding is in agreement with the previous studies. Wikandari et al. [20] reported that the chilli from the traditional market was heavily contaminated with fungi. The fungal counts ranged from 3 to 5.61 log CFU/g. Temu [33] reported that chilli and black pepper were the most contaminated samples, while curry powder was less contaminated by fungi. From our study, curry powder was recorded as one of the highly contaminated samples. Corroborating our study, Makhlouf et al. [3] stated that mixed spices such as curry powder and korma powder might be exposed to numerous fungi because they consist of various spices, including coriander seeds, cumin, white pepper, etc.. Different types of spices might be contaminated with varying sources of contamination, and this might be the primary source of the high fungal load in mixed spices.

In the case of whole spices, cinnamon and fennel had the lowest incidence of fungi, while the cloves and star anise were free from fungal contamination. The current findings are in line with the previous studies that reported low levels of fungal contamination in these spices [1,21,33]. The presence of antimicrobial compounds such as cinnamaldehyde, eugenol and anethole in these spices could be the reason for the low occurrence of fungi in these samples. According to Tajkarimi et al. [34], these compounds were proven to have an antimicrobial effect that prevented the growth of fungi in the spices.

According to Mandeel [11], the prevalence of fungi could be different among the spices due to the sample and sampling variability, the diverse processing, the storage and the place of origin. Essentially, the processing of spices will affect the fungal load in the samples. For example, the reduced contamination in white pepper compared to that in black pepper in this study might be attributed to the removal of the pericarp of white pepper during processing. This process will reduce the contamination by the fungi present in the pericarp, thus reducing fungal infection in white pepper compared to black pepper [8]. Instead, the wrinkled surface of black pepper is the ideal condition for fungal growth, thus resulting in a high percentage of fungal infection [35]. Besides that, the application of food irradiation could also reduce fungal contamination in spices. The irradiation process has been proven to be a safe and effective technique to get rid of fungi and maintain the nutrient benefit in foods [1,36].

Additionally, the spice production process, such as drying and packaging, may also affect the level of fungal contamination in spices. Spice-based products produced by large companies in Malaysia that were certified with MeSTI, HALAL, HACCP and GMP usually have a lower risk of fungal contamination. These certifications are important to ensure the quality and safety of the products. *Jabatan Kemajuan Islam Malaysia* (JAKIM) [37] has stated that any company that produces halal-certified products must comply with basic hygiene during the preparation, handling, processing, packaging, storage or transfer of the products. Thus, we can presume that the reason for the low fungal contamination might be due to the hygiene practices by the manufacturers. A recent study by Ahmad-Zaidi et al. [19] reported that the manufacturer’s size and certifications affected the level of fungal contamination and mycotoxins in spices and spice-based products. According to the authors, spice samples from small-scale manufacturers contain a high total fungal load compared to those from large-scale manufacturers, which usually adopt various food safety and quality certifications. In the case of whole spices, the susceptibility to fungal infection differs among the spices. Different matrices of the spices will have different sensitivities towards the fungi, thus affecting the fungi infection in the spices [1,38].

*Aspergillus* spp. and *Penicillium* spp. were the most frequent genera isolated from all of the samples from this study. The high frequencies of these genera indicated that the contamination might occur from the storage, as these genera are commonly found in low-moisture crops during storage. Our findings were in line with previous studies that reported the predominance of these two genera in spices [13,39,40,41].

*Aspergillus* spp.—mainly, *A. flavus* and *A. niger*—were present in almost all of the ground spice samples. *A. niger* was present in all of the whole spice samples except for cloves and star anise. This finding is in agreement with a previous work by Kulshrestha et al. [42]. The authors found that *Aspergillus* spp.—particularly, *A. flavus* and *A. niger*—were the most isolated species from the coriander, fennel and cumin samples in India. In Tanzania, Temu [33] isolated *A. niger* and *A. flavus* from black pepper, cinnamon, coriander seeds, curry powder, red chilli powder and white pepper. The author also reported the absence of fungal species in cloves. A study in Saudi Arabia by Hashem and Alamri [13] found that *A. niger* and *A. flavus* contaminated 87% and 80% of the spices, respectively. The species of *A. tamarii*, *A. sydowii*, *A. terreus, A. chevalieri, Chaetomium* sp. and *Cladosporium* sp. were also isolated from their study. In Italy, Garcia et al. [1] found that *Cladosporium* sp. and *A. niger* were mostly isolated from cinnamon. A recent study by Tsehaynesh et al. [43] reported that the most common *Aspergillus* spp. isolated from pepper samples were *A. flavus*, *A. parasiticus* and *A. niger*. In contrast, *A. parasiticus* was not recovered from any spices in the present study. Nurtjahja et al. [21] reported that *A. flavus* and *A. chevalieri* were the most isolated species from spice samples in Indonesia.

*A. flavus* and *A. niger* are known to produce harmful mycotoxins such as AFs and OTA. From the screening of AF- and OTA-producing isolates, one isolate of *A. flavus* was capable of producing AF, and two isolates of *A. niger* were capable of producing OTA. Although the amounts of AF and OTA were not determined in this study, previous studies from various countries have reported varying amounts of AF and OTA depending on the types of spices. In the present study, mycotoxigenic fungi such as *A. flavus* and *A. niger* were the dominant species found in the samples showing a high total fungal count (ground spices) or a high incidence of fungal contamination (whole spices). For example, the highest isolation frequency of *A. flavus* was reported in ground samples with a high total fungal count, such as chilli powder, dried chilli and coriander. For the whole spices, *A. flavus* was dominant in samples that recorded a high incidence of fungal contamination, such as white pepper and black pepper. Meanwhile, *A. niger* was mainly isolated from korma powder, dried chilli and curry powder, which are also highly contaminated. The current finding is in line with a previous report by Jeswal and Kumar [44], who pointed out that *A. flavus* was the dominant species in almost all of the spices tested in their study. The authors also found that 45.4% of the *A. flavus* isolated from the spices was able to produce AF, ranging from 2.1–16.40 µg/kg. A study in Qatar by Hammami et al. [39] reported that black pepper recorded the highest level of total aflatoxins (84.09 µg/kg).

Prelle et al. [45] found that 23.8% of their samples, including chilli, mixed spices and peppers, were contaminated with OTA, ranging from 1.61 to 19.06 µg/kg. In addition, AFs and OTA also contaminate other spices such as cinnamon, cloves and coriander. In Malaysia, Jalili and Jinap [22] revealed that the occurrence of AFs and OTA in dried chilli ranged from 0.20 to 79.70 µg/kg and from 0.20 to 101.20 µg/kg, respectively. From a recent study in Indonesia, Wikandari et al. [20] examined dried chilli, and the authors found the incidence of AFB1 (39.30–139.50 µg/kg), AFB2 (2.60–33.3 µg/kg) and OTA (23.70–84.60 µg/kg). These results prove that these spices are very susceptible to aflatoxigenic and ochratoxigenic fungi.

Fungal contamination is the primary concern in the food industry, since it may lead to food deterioration and mycotoxin contamination. The high consumption of spices contaminated with *A. flavus* and *A. niger* may increase mycotoxin exposure to the consumers. Chilli is one of the largest consumed commodities, following black pepper [20,46]. In Malaysia, chilli is widely used in the local cuisines. Apart from chilli, mixed spices such as korma powder and curry powder are also used as the main ingredients in Malaysian cuisine, especially *nasi kandar,* which is popular in the northern part of Peninsular Malaysia. Chicken, fish and beef are usually cooked using korma powder and curry powder to produce a flavourful gravy served in restaurants or sold in the market as ready-to-eat food in cans or retort pouches.

## 5. Conclusions

In conclusion, spices marketed by retailers in Penang, Malaysia, are susceptible to contamination by the mycotoxigenic fungi. Out of 13 spices, 4 spices, including coriander seeds, curry powder, korma powder, chilli powder and dried chilli, were heavily contaminated by fungi, ranging from 2.59 to 3.51 log CFU/g. *A. flavus* and *A. niger* were the predominant mycotoxigenic fungi found in spices, indicating the risk of mycotoxin contamination. These findings suggest that the storage conditions at the retailers’ premises might favour fungal growth. Thus, to prevent fungal growth and subsequent mycotoxin contamination, all stakeholders in the spices supply chain, including the farmers, importers, manufacturers and retailers, must practice proper storage management. Besides that, the authority should also play an important role in mycotoxin management. More surveillance studies on spices should be conducted to assess the risk of mycotoxin exposure from consuming spices-based products in Malaysia. In addition, the public should be well informed about the danger of mycotoxin contamination by creating awareness via social media.

## Figures and Tables

**Table 1:** Total fungal load and isolation frequency of fungal species from ground spices.

Spices (Number of Samples)		Fennel (*n* = 5)	White Pepper (*n* = 5)	Cinnamon (*n* = 5)	Cumin (*n* = 5)	Coriander (*n* = 5)	Black Pepper (*n* = 5)	Curry Powder (*n* = 5)	Korma Powder (*n* = 5)	Dried Chilli (*n* = 5)	Chilli Powder (*n* = 5)	Chilli Paste (*n* = 5)
Water activity		0.615	0.620	0.634	0.623	0.614	0.613	0.500	0.643	0.621	0.592	0.955
Total fungal count (log CFU/g)	ADM	2.28 ± 0.48	2.23 ± 1.28	1.67 ± 0.94	2.40 ± 0.32	3.08 ± 0.33	1.95 ± 1.17	3.17 ± 0.45	2.59 ± 0.16	3.51 ± 0.92	2.59 ± 1.16	0.71 ± 1.31
	DG18	2.31 ± 0.50	1.82 ± 1.68	2.30 ± 0.18	2.22 ± 1.37	3.14 ± 0.26	2.95 ± 0.44	2.80 ± 0.75	2.65 ± 0.54	1.67 ± 1.47	2.25 ± 1.69	0.45 ± 0.76
Fungal species		IF * (%)	IF (%)	IF (%)	IF (%)	IF (%)	IF (%)	IF (%)	IF (%)	IF (%)	IF (%)	IF (%)
*A. aculeatus*		-	-	-	-	-	-	-	-	1.5	-	-
*A. amstelodami*		-	-	-	7.1	4.2	-	-	20.0	-	-	-
*A. chevalieri*		-	-	6.5	-	1.1	12.0	3.0	-	-	-	-
*A. flavus*		15.7	40.0	9.7	14.3	31.6	1.4	-	2.5	45.5	76.3	-
*A. niger*		17.6	-	6.5	17.9	10.5	9.6	24.2	38.0	25.8	0.2	3.3
*A. sydowii*		-	-	-	-	1.1		1.5	2.5	-	-	-
*A. tamarii*		-	-	6.5	-	1.1	12.3	1.5	2.5	-	-	-
*A. terreus*		-	1.3	-		2.1	4.1	-		-	-	1.2
*P. chrysogenum*		-	-	-	7.1	10.5	-	-	7.5	-	0.2	-
*P. citrinum*		-	-	-	-	1.1	-	-	2.5	-	-	-
*P. hispanicum*		-	-	-	-	-	-	-	-	-	-	-
*Chaetomium jabalpurense*		9.8	13.3	-	14.3	-	-	-	-	-	-	-
*Cladosporium tenuissimum*		-	-	71.0	-	-	5.5	4.6	-	-	-	-

* Isolation frequency.

**Table 2:** Incidence of fungal infection and isolation frequency of fungal species from whole spices.

Spices (Number of Samples)	Fennel (*n* = 5)	White Pepper (*n* = 5)	Cinnamon (*n* = 5)	Cumin (*n* = 5)	Coriander (*n* = 5)	Black Pepper (*n* = 5)	Star Anise (*n* = 5)	Cloves (*n* = 5)
Water activity	0.638	0.635	0.657	0.642	0.711	0.630	0.601	0.625
Incidence of fungal contamination (%) (Range)	52.0 ± 26.4 (27–85)	83.2 ± 23.0 (56–100)	30.6 ± 41.3 (0–100)	56.4 ± 27.9 (13–82)	92.4 ± 11.8 (73–100)	94.0 ± 13.4 (70–100)	0 0	0 0
Fungal species	IF (%)	IF (%)	IF (%)	IF (%)	IF (%)	IF (%)	IF (%)	IF (%)
*A. aculeatus*	-	-	-	-	-	-	-	-
*A. amstelodami*	-	-	16.7	8.6	33.3	6.4	-	-
*A. chevalieri*	-	5.0	-	2.9	-	4.3	-	-
*A. flavus*	-	70.0	-	2.9	7.1	34.0	-	-
*A. niger*	33.3	17.5	33.3	5.7	14.3	27.7	-	-
*A. sydowii*	25.0	15.0	-	20.0	7.1	6.4	-	-
*A. tamarii*	-	-	-	-	-	-	-	-
*A. terreus*	-	-	-	-	-	-	-	-
*P. chrysogenum*	-	-	-	5.7	14.3	-	-	-
*P. citrinum*	8.3	-	-	-	11.9	4.3	-	-
*P. hispanicum*	-	-	58.3	-	-	-	-	-
*Chaetomium jabalpurense*	20.8	-	-	5.7	4.8	-	-	-
*Cladosporium tenuissimum*	-	-	-	2.9	-	-	-	-

**Table 3:** Fungal identification based on the ITS region and detection of AF and OTA in the representative fungal isolates.

Isolate no.	Species	GenBank (% Similarity)	Source	Mycotoxin-Producing Ability (Green Fluorescence Intensity)	
				AF	OTA
A6	*A. niger*	100	Fennel	–	+
A2	*A. chevalieri*	100	White pepper	–	–
A	*A. terreus*	100	White pepper	–	–
A5	*A. flavus*	100	White pepper	++++	–
A10	*A. chevalieri*	100	Coriander	–	–
A12	*A. sydowii*	100	Coriander	–	–
C18	*Cladosporium tenuissimum*	100	Coriander	–	–
P23	*P. citrinum*	100	Coriander	–	–
A30	*A. chevalieri*	100	Coriander	–	–
C33	*Chaetomium jabalpurense*	100	Coriander (whole)	–	–
A8	*A. chevalieri*	100	Cumin	–	–
P19	*P. citrinum*	100	Cumin (whole)	–	–
A25	*A. chevalieri*	100	Cumin	–	–
A26	*A. sydowii*	100	Cumin (whole)	–	–
P31	*P. chrysogenum*	99	Cumin	–	–
A7	*A. tamarii*	100	Curry powder	–	–
A15	*A. sydowii*	99	Curry powder	–	–
A21	*A. chevalieri*	100	Curry powder	–	–
A9	*A. flavus*	100	Dried chilli	–	–
A20	*A. chevalieri*	99	Chilli powder	–	–
A22	*A. niger*	100	Dried chilli	–	+
A29	*A. aculeatus*	100	Dried chilli	–	–
P13	*P. hispanicum*	100	Cinnamon (whole)	–	–
A16	*A. chevalieri*	100	Black pepper	–	–

(+) low intensity, (++++) high intensity, (–) not detected.

## Data Availability

The data presented in this study are available on request from the corresponding author.
